# Asymmetric surge optimization during peak seasons: a discrete-event simulation of centralized fast-track operating rooms at a China National Children's Medical Center

**DOI:** 10.3389/fped.2026.1835090

**Published:** 2026-07-08

**Authors:** Yu Chen, Weihong Xu, Nanping Shen, Jiwen Sun, Bin Ji, Siyuan Wang, Wei Chen

**Affiliations:** 1 Department of Anesthesiology, Shanghai Children’s Medical Center, Shanghai Jiao Tong University School of Medicine, Shanghai, China; 2 Department of Nursing, Shanghai Children’s Medical Center, Shanghai Jiao Tong University School of Medicine, Shanghai, China

**Keywords:** discrete event simulation, hospital management, operating room efficiency, pediatric surgery, surgery schedule management

## Abstract

**Background:**

Holiday-related effects result in a substantial surge in the volume of pediatric surgeries. Managing operating room (OR) capacity during summer holiday is immensely challenging for a China National Children's Medical Center. A surge of pediatric surgeries is inherently asymmetric, characterized by a disproportionate increase in short elective procedures, while the volume of complex surgeries remains relatively stable. Traditional mixed-acuity scheduling or naive departmental segregation often results in severe capacity fragmentation. This study employs discrete-event simulation (DES) to propose and evaluate an asymmetric, centralized fast-track OR allocation strategy.

**Methods:**

Using retrospective, de-identified operational data from Shanghai Children's Medical Center, a National Children's Medical Center in China, we constructed a DES model representing the workflow of diverse surgical departments. Following a retrospective iterative calibration ensuring <2% baseline error, we introduced a Prospective Simulation Probing (PSP) mechanism under a dynamic safety-buffer policy to determine variance-penalized daily capacities. In the primary optimized scenario, fast-track turnover was modeled as Triangle (10, 12.5, 15) minutes rather than regular-OR Triangle (25, 27.5, 30) minutes, corresponding to a 15-minute mean reduction; 5- and 10-minute reduction scenarios were tested separately. OR days were first allocated to secure historical complex surgery volumes, after which residual days were pooled into a centralized fast-track center. Uncertainty was summarized as mean, SD, median, and 95% simulation intervals; sensitivity analyses varied turnover reduction, bounded fast-track demand, and edge-case fast-track classification.

**Results:**

Simulation revealed that simplistic departmental segregation penalized low-volume specialties, reducing their throughput due to idle-time waste. Conversely, the asymmetric centralized pooling model protected severe, slow-track case throughput while increasing fast-track capacity. The optimized 15-minute turnover-reduction scenario yielded a mean total throughput of 3,588.7 cases (median 3,589; 95% simulation interval, 3,562–3,614), corresponding to a 38.29% increase over the historical baseline of 2,595 cases (95% interval for increase, 37.26%–39.27%). Under the more conservative 10-minute reduction scenario, mean throughput was 3,208.3 cases, a 23.63% increase.

**Conclusions:**

DES modeling suggests that overcoming pediatric surgery surge requires separating short fast-track procedures from complex surgeries while explicitly protecting complex-care access. Consolidating short procedures into dedicated, homogeneous ORs can reduce non-operative turnover waste and increase throughput; however, implementation depends on staffing flexibility, sufficient fast-track demand, and local cost considerations.

## 1 Introduction

Operating rooms (ORs) are the most resource-intensive and revenue-generating units in healthcare facilities (1, 2). In China, National Children's Medical Centers face extreme operational stress during the “summer surge”—a highly concentrated period of healthcare demand coinciding with school holidays (3, 4). Crucially, this surge is fundamentally asymmetric: the volume of short, elective fast-track procedures (e.g., circumcisions, adenoidectomies) skyrockets (5), whereas the demand for complex, slow-track surgeries (e.g., neurosurgical tumor resections) remains relatively stable.

Traditional OR scheduling relies on mixed-acuity pooling, where long and short surgeries are interleaved. This approach allows surgeons to intuitively use short cases to fill residual fragmented time—a phenomenon often described as the “sand-and-rock” principle. However, this intuitive scheduling inherently limits turnover efficiency (6), as the unpredictable completion times of complex surgeries create frequent idle periods that short cases cannot fully utilize. Consequently, administrators often attempt to establish dedicated “fast-track” ORs to reduce turnover times (7). However, implementing such dedicated rooms within rigid departmental boundaries—often referred to as “departmental silos”—can yield counterintuitive results. Low-volume specialties may experience a “capacity fragmentation penalty”: the efficiency gained from faster turnover in dedicated fast-track rooms is outweighed by the loss of opportunities to fill end-of-day gaps in regular mixed-acuity ORs.

While previous literature has utilized Discrete-event Simulation (DES) to optimize OR schedules, most studies rely on static arithmetic heuristics that fail to account for the high variance inherent in complex surgeries (8–12). Alternatively, some enforce strict proportional scaling, which artificially constrains efficiency gains. To address this literature gap, we developed a highly robust DES framework to evaluate an Asymmetric Surge SLA-Maximization strategy.

We hypothesized that breaking departmental silos to create a centralized fast-track OR pool—while strictly safeguarding complex surgery volumes through Service Level Agreements (SLAs) (13)—would maximize global throughput without distorting case-mix integrity.

## 2 Methods and materials

### 2.1 Setting and model perspectives

This retrospective operational modeling study was conducted at Shanghai Children's Medical Center, a tertiary first-class hospital and one of China's National Children's Medical Centers, with 1,500 beds and 25 operating rooms and an annual surgical volume of about 30,000 cases. The analysis used de-identified operating-room log data collected between August 3, 2025, and August 29, 2025, covering 20 working days during the summer peak period. The modeled setting was the non-cardiothoracic pediatric surgery building, which included 15 fully staffed elective operating rooms across multiple surgical departments (e.g., orthopedics, urology, general surgery, neurosurgery, dermatology), each operating for 10 h per working day. Appendix describes the estimated overall operating room capacity and utilization in more detail. The operating room nursing department is responsible for perioperative care and logistical activities, such as patient transfer, surgical instruments, and operating room cleaning (14). At Shanghai Children's Medical Center, each surgical department is allocated a certain number of operating rooms per day per week (Table 1). The hospital maintains dedicated operating rooms for non-elective procedures; consequently, the ORs included in this study were rarely used for emergency or add-on cases. All operating rooms opened daily at 8:00 am. The model output was operating room throughput during the observation period, and scenario analysis was used to evaluate the optimization scheme.

**Table 1:** Number of ORs designated to each department on each workday.

Workday	Burn Surgery	Dermatology	ENT^a^	General Surgery	Neurosurgery	OMFS^b^	Ophthalmology	Orthopedics	Urology
Mon	1	0	2	2	2	1	1	3	3
Tue	0	1	2	2	1	0	2	3	4
Wed	1	0	2	2	2	1	1	3	3
Thu	0	2	2	2	1	0	1	3	4
Fri	1	0	2	2	2	1	1	3	3

a ENT, Ears, Nose, and Throat/Otorhinolaryngology.

b OMFS, Oral and Maxillofacial Surgery.

### 2.2 Model structure and iterative calibration

This model simulates the perioperative pathway of inpatient surgical cases on the day of surgery, spanning from preoperative preparation to the completion of OR turnover for the subsequent patient. Daily active ORs were parameterized according to Table 1. Because the observed summer period had a substantial elective backlog, the model drew patients from an inexhaustible queue in the primary analysis; bounded-demand sensitivity analyses were then used to evaluate the effect of this assumption. For each simulated entity, the surgical procedure type was stochastically assigned using the sample() function in R, weighted by historical proportional frequency. No new case could start after the dynamically determined cut-off time, although ongoing surgeries were allowed to finish. Post-anesthesia care unit capacity was not modeled as a restrictive bottleneck because the center operates a scalable PACU buffering area; this assumption is addressed in the limitations. The simulation was implemented in R 4.6.0 using the simmer package 4.4.7.

### 2.3 Data inputs

Real-world operative time data for non-cardiothoracic pediatric surgeries were collected between August 3, 2025, and August 29, 2025, and stratified by surgical department and procedure type. Designated nursing staff recorded the exact operating room entry and exit times for each patient, along with the specific surgical procedure performed. Empirical procedure-level 95th percentiles were calculated to support fast-track classification, and procedure durations were then simulated using truncated lognormal distributions parameterized by empirical mean, SD, minimum, and maximum values. Parameters governing the remaining perioperative workflow stages were derived from structured interviews with the head OR nurse and are reported in the Appendix.

### 2.4 Model verification and validation

**Figure 1:**
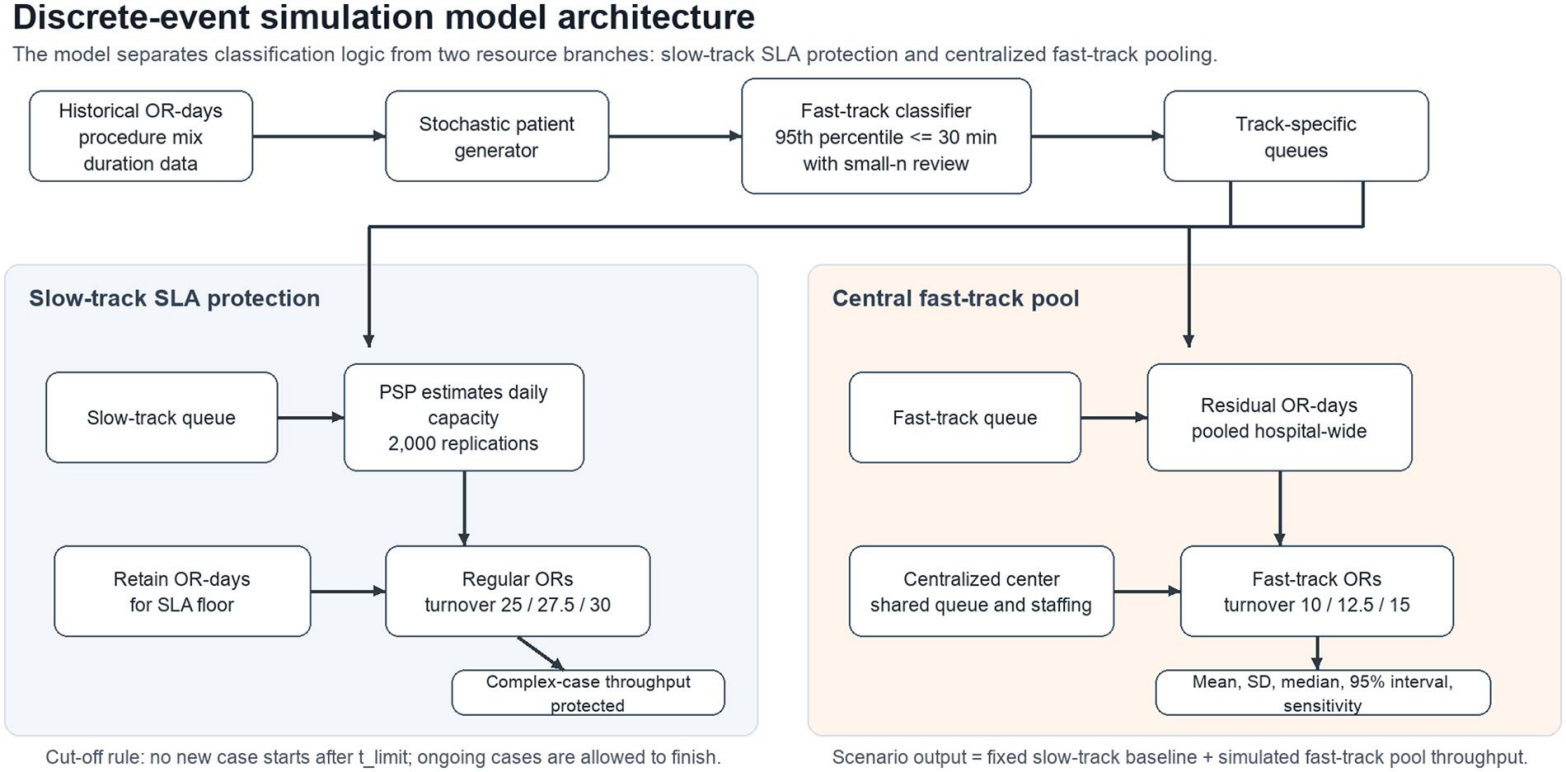
Discrete-event simulation model architecture. The model uses empirical procedure mix, stochastic patient generation, track-specific queues, explicit OR-day cut-offs, and separate slow-track SLA and centralized fast-track resource branches.

**Figure 2:**
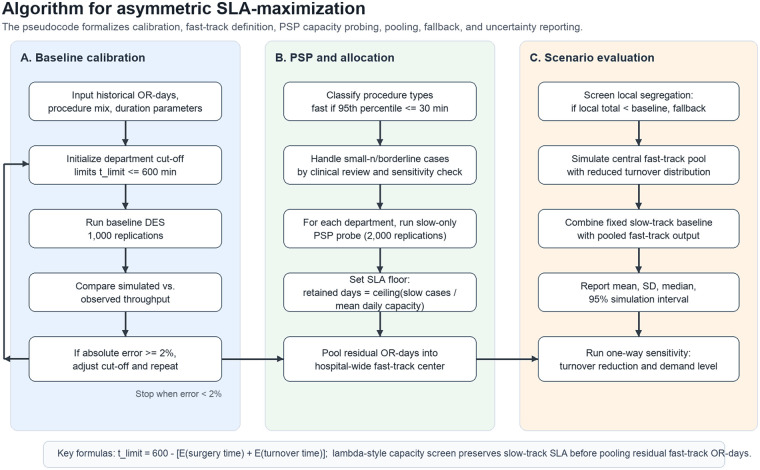
Algorithmic flowchart for baseline calibration, fast-track classification, PSP capacity probing, centralized pooling, baseline fallback screening, and uncertainty reporting.

The DES architecture is depicted in Figure 1, and the algorithmic flowchart is depicted in Figure 2. To ensure robustness, the framework underwent a two-step validation process: face validation via expert consultation with the head OR nurse, and empirical validation by benchmarking simulated outputs against historical real-world data. Baseline validation used 1,000 replications per department; the optimized PSP and fast-track pooling analyses used 2,000 replications because they determine the SLA floor and pooled-center capacity. These replication counts produced Monte Carlo standard errors below one aggregate case for total-throughput estimates, while keeping the simulation computationally practical. For every stochastic output, we report the mean, SD, median, and the 2.5th−97.5th percentile simulation interval.

### 2.5 Scenario analysis: centralized fast-track pooling and asymmetric optimization

To address the severe capacity fragmentation inherent in traditional mixed-acuity scheduling, we designed an Asymmetric SLA-Maximization scenario. In plain terms, this strategy first protects the historical volume of complex, slow-track cases and then uses only the residual OR-days to maximize fast-track capacity through a centralized pool. PSP denotes the repeated prospective simulation probe used to estimate slow-track daily capacity before allocation. Limiting-Factor Proportional Scaling denotes the conservative capacity screen used to prevent a throughput-maximizing solution from eroding the complex-care mandate.

#### 2.5.1 Definition and operational mechanism of fast-track surgeries

First, we established criteria for segregating surgical workflows. Fast-track surgeries were defined as procedure types with short empirical operative-time distributions, primarily those with a 95th percentile of real-world operative time ≤30 min. Edge cases were defined as small-sample procedures (*n* < 30) whose observed operative time extended beyond the 30-minute threshold (maximum >30 min), so that their fast-track classification was statistically uncertain; these were reviewed with surgical and OR nursing leadership, explicitly flagged in Table 2, and stress-tested in an exclusion sensitivity analysis. By contrast, high-volume procedures (*n* ≥ 30) were classified confidently from their P95 despite occasional long outliers, and small-sample procedures bounded at or below 30 min were treated as unambiguously fast-track. All other procedures were classified as complex, slow-track surgeries. The primary optimized scenario modeled a homogeneous fast-track setting with OR turnover time Triangle (10, 12.5, 15) minutes, compared with Triangle (25, 27.5, 30) minutes in regular ORs. This primary assumption represents a 15-minute mean turnover reduction; 5- and 10-minute reductions were analyzed as conservative sensitivity scenarios.

**Table 2:** Characteristics of fast-track surgeries included in the centralized pool. P95 denotes the empirical 95th percentile of observed operative time. Edge cases are small-sample procedures (*n* < 30) whose observed maximum exceeds 30 min; they were retained in the clinical fast-track set after review with surgical and OR nursing leadership and were checked in an exclusion sensitivity analysis. A source-data audit confirmed that neurosurgery biopsy had a maximum observed duration of 40 min; the earlier 140-minute value in the original supplementary table was corrected as a transcription error.

Department	Fast-track procedure	Cases	Mean	SD	Min	P95	Max	Handling
ENT	Adenoidectomy	13	12.8	7.7	5	25.0	25	Primary
Neurosurgery	Biopsy	8	24.4	6.8	20	34.8	40	Edge case
General Surgery	Branchial cleft fistula excision	3	26.7	10.4	15	34.5	35	Edge case
Ophthalmology	Chalazion curettage	45	12.9	7.6	5	29.0	35	Primary
Urology	Circumcision	433	6.0	3.3	5	15	28	Primary
Neurosurgery	Hematoma drainage	10	14.0	9.9	5	30.5	35	Edge case
General Surgery	Labial Adhesion Surgery	26	5.4	1.4	5	8.7	10	Primary
OMFS	Lingual frenulum lengthening	15	11.7	6.5	5	23.0	30	Primary
Orthopedics	Quick internal fixation removal	116	9.1	5.5	4	20.0	30	Primary
Burn Surgery	Scar contracture release	32	9.7	7.1	6	19.5	40	Primary
General Surgery	Sclerotherapy for lymphatic malformation	10	18.0	8.6	5	30.0	30	Primary
Dermatology	Skin lesion excision	28	18.2	8.2	5	30.0	35	Edge case
General Surgery	Urachal cyst excision	20	8.2	4.4	6	15.3	20	Primary
ENT	Uvulopalatopharyngoplasty	375	14.0	9.2	5	30.0	75	Primary
TOTAL		1,134						

#### 2.5.2 Securing complex care via prospective simulation probing

A primary risk in throughput-maximizing models is the neglect of complex surgeries. To prevent this, our algorithm imposed a strict Service Level Agreement (SLA) floor: every department retained enough regular OR-days to reproduce its historical slow-track volume before any residual OR-days were pooled. The PSP probe ran 2,000 replications for each department's slow-track case mix, extracted the mean daily slow-track capacity together with SD, median, and 95% simulation interval, and set retained OR-days as ceiling (historical slow-track cases/mean daily slow-track capacity). The start cut-off formula was t_limit = 600 − [E(surgery time) + E(turnover time)], while stochastic variance entered through the full replication distribution and the retained-day rounding step rather than through a deterministic arithmetic average alone.

#### 2.5.3 Departmental capacity reallocation and centralized pooling

After securing the SLA floor, all remaining OR days previously allocated to individual departments were reallocated and aggregated into a single, hospital-wide Centralized Fast-Track Pool. This paradigm shift resolves a critical temporal demand mismatch. In siloed departments, patients requiring fast-track surgeries often face prolonged batch-waiting because their specific department might only justify one fast-track OR day per month. By pooling surrendered days centrally, the hospital maintains continuously active fast-track ORs every day. This scheduling liquidity ensures that fast-track cases from any department can be absorbed promptly, perfectly aligning OR availability with the continuous daily arrival of patients during summer surges.

#### 2.5.4 Proportional optimization and baseline fallback

Finally, the pooled fast-track days were simulated to process the hospital-wide backlog of fast-track surgeries. To reduce case-mix distortion, the algorithm retained the slow-track SLA floor before allocating residual OR-days to the centralized fast-track center. Let lambda_fast denote simulated fast-track output divided by historical fast-track volume and lambda_slow denote protected slow-track output divided by historical slow-track volume; the effective scale factor was lambda = min(lambda_fast, lambda_slow), so any scenario with lambda_slow < 1 was rejected before fast-track gains were counted. Scenario outputs were then reported as the fixed historical slow-track baseline plus the simulated pooled fast-track output, with uncertainty intervals and sensitivity analyses.

Furthermore, the algorithm incorporated a baseline fallback screen. For each department, we compared the mean throughput under local departmental fast-track segregation with that department's historical baseline. If local segregation underperformed baseline, the department was marked as a fallback case and the result was interpreted as evidence that local segregation alone would be counterproductive; these departments motivated the centralized pooling strategy rather than siloed fast-track rooms.

Algorithm 1. **Asymmetric SLA-Maximization pseudocode**

1. Input historical OR-day allocation, department, procedure type, operative duration, and turnover distributions.

2. For each department, initialize the baseline start cut-off t_limit ≤600 min.

3. Run 1,000 baseline DES replications and compare mean simulated throughput with observed throughput.

4. If absolute error is ≥2%, adjust t_limit in 1-minute increments toward the observed volume and repeat until error <2% or 50 calibration iterations are reached.

5. Compute procedure-level empirical 95th percentile operative time and classify fast-track candidates using P95 ≤30 min as the primary rule.

6. Flag edge-case candidates—small-sample procedures (*n* < 30) whose observed maximum exceeds 30 min—for clinical review and an edge-case exclusion sensitivity analysis, and classify the remaining procedures by P95.

7. For each department, remove fast-track candidates and run a 2,000-replication slow-track PSP probe.

8. Extract mean daily slow-track capacity, SD, median, and 95% simulation interval.

9. Set retained OR-days = ceiling(historical slow-track cases/mean daily slow-track capacity).

10. Aggregate residual OR-days across departments into a centralized fast-track pool.

11. Compute lambda_fast, lambda_slow, and lambda = min(lambda_fast, lambda_slow); reject any allocation with lambda_slow <1.

12. Screen local departmental segregation; if local total throughput is below baseline, mark baseline fallback.

13. Simulate the centralized fast-track pool with reduced turnover and report mean, SD, median, and 95% simulation interval for fast-track and total outputs.

14. Repeat scenario runs under turnover-reduction, bounded-demand, and edge-case classification sensitivity settings.

## 3 Results

### 3.1 Model verification and validation

According to the head nurse's expert assessment, the generalized OR workflow captured actual operational practices. After verification, the baseline workflows for all nine surgical departments were simulated 1,000 times and compared with actual August 2025 throughput. Across the hospital cohort, the simulated mean was 2,606.7 cases (SD 25.3; median 2,607; 95% simulation interval, 2,558–2,654), a 0.45% deviation from the observed 2,595 cases. Department-level deviations were all within approximately 2% (Table 3), indicating satisfactory calibration.

**Table 3:** Model validation results for the baseline scenario.

Department	Real data	Mean (SD)	Median	95% SI	Deviation (%)	Cut-off (min)
BurnSurg	122	123.9 (5.4)	124	113–134	1.54	565.4
Dermatology	107	107.3 (3.7)	107	100–115	0.28	575.6
ENT	442	447.8 (8.0)	448	432–462	1.32	486
General	288	290.7 (9.9)	291	272–310	0.94	512.6
Neuro	133	135.2 (6.0)	135	123–148	1.62	441.7
OMFS	96	96.1 (3.4)	96	90–103	0.14	556
Ophtha	218	218.3 (4.7)	218	209–227	0.12	536.9
Ortho	433	434.3 (13.4)	434	409–461	0.30	483
Urology	756	753.2 (13.7)	754	724–779	−0.37	506
TOTAL	2,595	2,606.7 (25.3)	2,607	2,558–2,654	0.45	NA

### 3.2 Scenario analysis results

**Figure 3:**
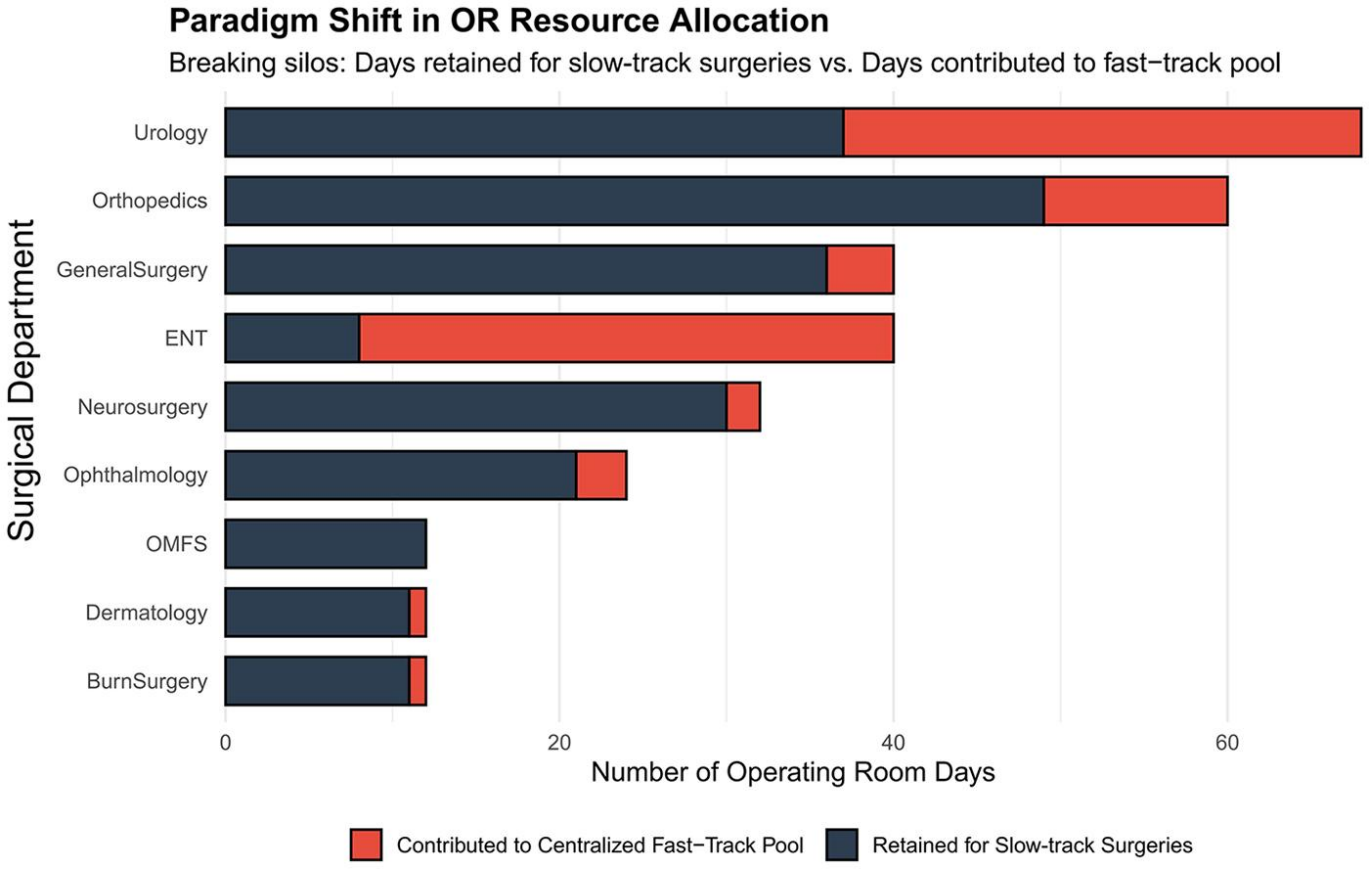
Paradigm shift in OR resource allocation.

**Figure 4:**
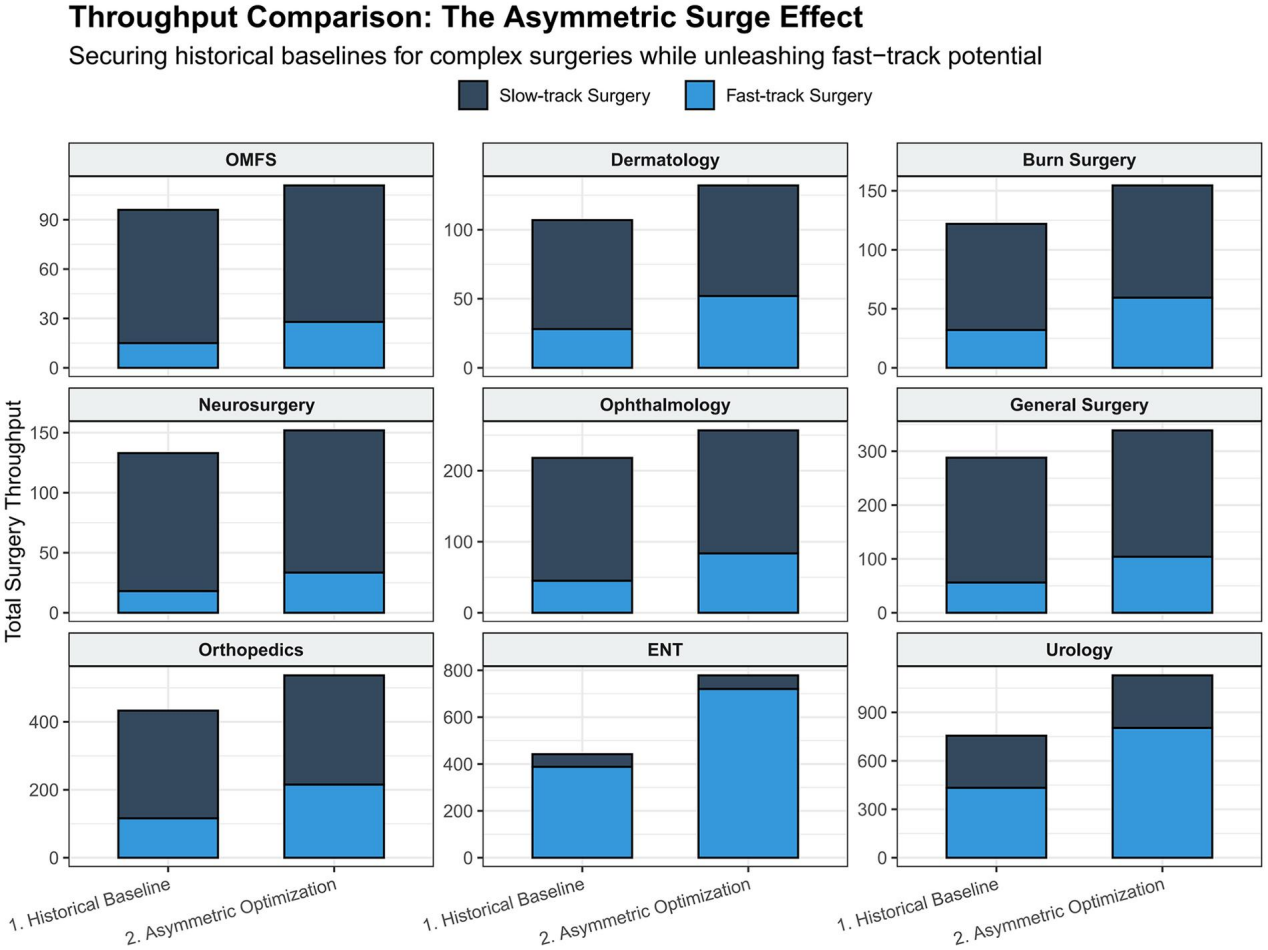
Throughput comparison of historical baseline and asymmetric optimization.

The Asymmetric SLA-Maximization scenario bypassed the fragmentation penalty. By retaining the OR-days required for slow-track cases and pooling 86 residual fast-track OR-days, the optimized 15-minute turnover-reduction scenario increased mean total throughput from the observed baseline of 2,595 cases to 3,588.7 cases (SD 13.5; median 3,589; 95% simulation interval, 3,562–3,614), corresponding to a mean increase of 38.29% (95% simulation interval for increase, 37.26%–39.27%) (Table 4). Slow-track complex surgery volume was fixed at its historical baseline of 1,461 cases in the optimized calculation, while the pooled fast-track component had a mean output of 2,127.7 cases (SD 13.5; median 2,128; 95% simulation interval, 2,101–2,153). At a more conservative 10-minute turnover reduction, mean total throughput was 3,208.3 cases, corresponding to a 23.63% increase; under bounded fast-track demand, gains fell to 10.92%–21.85%. Excluding all edge-case fast-track procedures did not erode the conclusion (mean total throughput, 3,668.9; 95% simulation interval, 3,642–3,694; increase, 41.38%). Figure 3 illustrates resource reallocation, and Figure 4 shows the asymmetric throughput increase. The local segregation screen showed that Burn Surgery, Dermatology, and OMFS triggered baseline fallback because local dedicated fast-track rooms produced lower mean throughput than historical mixed scheduling (Table 5). In the final centralized model, fast-track cases from every department—including these three fallback specialties—are absorbed by the shared pool, so the department-level gains shown in Figure 4 are realized hospital-wide; the fallback screen indicates only that isolated, department-level segregation would have been counterproductive.

**Table 4:** Optimized scenario uncertainty and sensitivity analyses.

Analysis	Setting	Mean	SD	Median	95% SI	Increase
Primary scenario	Total optimized output; 86 pooled OR-days; 15-min turnover reduction	3,588.7	13.5	3,589	3,562–3,614	38.29% (37.26–39.27%)
Output component	Fixed slow-track/complex throughput	1,461	0	1,461	1,461–1,461	Preserved baseline
Output component	Pooled fast-track throughput	2,127.7	13.5	2,128	2,101–2,153	Component output
Turnover sensitivity	5-min reduction	2,941.3	8.3	2,941	2,925–2,957	13.35%
Turnover sensitivity	10-min reduction	3,208.3	10.6	3,209	3,187–3,229	23.63%
Turnover sensitivity	15-min reduction	3,588.7	13.5	3,589	3,562–3,614	38.29%
Demand sensitivity	1.25× historical fast-track demand	2,878.5	0	2,878	2,878–2,878	10.92%
Demand sensitivity	1.50× historical fast-track demand	3,162	0	3,162	3,162–3,162	21.85%
Demand sensitivity	2× historical fast-track demand	3,588.7	13.5	3,589	3,562–3,614	38.29%
Classification sensitivity	Edge-case procedures excluded	3,668.9	13.3	3,669	3,642–3,694	41.38%

**Table 5:** Baseline fallback screen for local departmental fast-track segregation.

Department	Baseline	Local segregated mean	Delta	Fallback?	Reason
Burn Surgery	122	114.7	−7.3	Yes	Local segregation below baseline
Dermatology	107	98.7	−8.3	Yes	Local segregation below baseline
ENT	442	767.6	325.6	No	Local segregation above baseline
General Surgery	288	334.7	46.7	No	Local segregation above baseline
Neurosurgery	133	170.0	37.0	No	Local segregation above baseline
OMFS	96	81	−15	Yes	Local segregation below baseline
Ophthalmology	218	242.2	24.2	No	Local segregation above baseline
Orthopedics	433	610.0	177.0	No	Local segregation above baseline
Urology	756	1,212.6	456.6	No	Local segregation above baseline

## 4 Discussion

Our simulation framework yields several insights for healthcare operations management: overcoming pediatric summer surges does not demand the sacrifice of complex care, nor does it necessarily require expanding physical infrastructure. Rather, it necessitates a structural evolution from siloed departmental scheduling to algorithmically driven, globally pooled resource management. A key finding of this study is the quantitative demonstration of the “sand-and-rock” phenomenon. In traditional scheduling, surgeons intuitively use short fast-track surgeries (“sand”) to fill the fragmented time left by complex surgeries (“rocks”) (15). Stripping away these short procedures into dedicated rooms within small departments creates severe end-of-day idle waste.

Our model demonstrates that fast-track segregation requires a critical mass of volume to be efficient; otherwise, preserving the natural pooling efficiency of mixed-acuity scheduling remains superior. The terms used in the model have practical meanings: Asymmetric SLA-Maximization means protecting complex-care access before maximizing fast-track volume; PSP is a prospective simulation probe of daily slow-track capacity; and Limiting-Factor Proportional Scaling is the conservative screen that prevents the fast-track gain from overriding the slow-track SLA floor. This approach confirms that efficiency gains do not require compromising a national medical center's primary responsibility to treat complex, severe diseases.

Furthermore, this study highlights the advantages of stochastic DES over static arithmetic heuristics in healthcare resource planning. Static formulas systematically underestimate the variance penalty introduced by long complex surgeries, leading to catastrophic queuing deadlocks in real-world applications.

Limitations: This single-center, retrospective operational model depends on several implementation assumptions. First, the centralized fast-track pool assumes that OR nursing and anesthetic staff can be redeployed across specialties after appropriate credentialing and cross-training; real-world implementation would require staffing, logistics, and cost analysis. Second, the primary model assumes a sufficiently large summer backlog to saturate fast-track capacity. Bounded-demand sensitivity analyses showed that throughput gains fell to 10.92% and 21.85% when fast-track demand was capped at 1.25× and 1.50× historical fast-track volume, respectively. Third, PACU capacity was not modeled as a bottleneck based on local operational practice; future work should incorporate PACU census data where recovery capacity is constrained. Fourth, the primary turnover-time reduction was a 15-minute expert operational estimate rather than a prospective measurement; the 10-minute sensitivity scenario still increased mean throughput by 23.63%, but the magnitude of benefit is clearly sensitive to this assumption. Finally, this study measured throughput and resource-use effects only; it did not perform cost-effectiveness, staffing-cost, or return-on-investment analysis, so economic claims should be interpreted cautiously.

## 5 Conclusion

This study developed a DES model to optimize OR allocation during peak pediatric surgery periods. The results suggest that overcoming the Capacity Fragmentation Penalty requires breaking departmental silos while explicitly protecting complex-care access. By securing slow-track surgery baselines and pooling residual resources into a centralized fast-track center, hospitals may increase peak-season throughput without compromising clinical care responsibilities, provided that local staffing, demand, PACU, and cost constraints are addressed.

## Data Availability

The original contributions presented in the study are included in the article/Supplementary Material, further inquiries can be directed to the corresponding author/s.
