# Supplementary material for: Asymmetric surge optimization during peak seasons: a discrete-event simulation of centralized fast-track operating rooms at a China National Children's Medical Center
Source: Front Pediatr. 2026 Jul 8;14:1835090. doi: 10.3389/fped.2026.1835090 (PMC13388461; doi:10.3389/fped.2026.1835090)
Supplement: Supplementary file 1 [file Datasheet1.docx]

**Appendix**

# Title: Asymmetric Surge Optimization During Peak Seasons: A Discrete-Event Simulation of Centralized Fast-Track Operating Rooms at a China National Children's Medical Center

Wei Chen*, Yu Chen

***Corresponding author**: Wei Chen, Department of Anesthesiology, Shanghai Children's Medical Center, School of Medicine, Shanghai Jiao Tong University, Shanghai 200030, China; [chenwei@scmc.com.cn](mailto:yihan2k5@gmail.com)

**Model setting**

*(1) Estimation of overall OR capacity and utilization during the study period*

This study was modeled based on the empirical operational data from a core facility of the China National Children's Medical Center. The observation period spanned from August 3, 2025, to August 29, 2025, capturing the extreme "summer surge" phenomenon typical in pediatric healthcare. During these 20 working days, 15 dedicated elective operating rooms (ORs) were fully staffed and operational for 10 hours (600 minutes) each day. This equated to a total capacity of 300 OR-days (or 180,000 OR minutes) during the study period. These 300 OR-days were historically allocated across nine surgical departments: Urology (68 days), Orthopedics (60 days), ENT (40 days), General Surgery (40 days), Neurosurgery (32 days), Ophthalmology (24 days), Burn Surgery (12 days), Dermatology (12 days), and Oral and Maxillofacial Surgery (OMFS) (12 days).

*(2) Non-elective surgeries*

The hospital operates a separate suite of ORs dedicated exclusively to trauma, urgent, and emergency procedures. Consequently, the 15 ORs investigated in this study were rarely interrupted by non-elective surgeries. Furthermore, during the summer peak season, elective OR utilization rates are exceptionally high across all departments. Based on these operational realities, non-elective or "add-on" emergency cases were not explicitly modeled in this simulation.

**Model structure**

The model simulates the perioperative pathway of inpatient and day-surgery patients on the day of surgery. Because top-tier national pediatric hospitals face an overwhelming backlog of elective patients during the summer holidays, the model assumes a continuous, inexhaustible patient pool (i.e., infinite queue).

For each patient entity generated in the simulation, a specific surgery type is stochastically assigned using the sample() function in R. This selection is weighted strictly by the historical proportional frequency (Patient %) of each procedure type within its respective department, ensuring absolute case-mix integrity.

Unlike previous models, Post-Anesthesia Care Unit (PACU) availability was not modeled as a restrictive bottleneck. The National Children's Medical Center operates an abundant, highly scalable PACU buffering zone. Therefore, the "Check PACU availability" and "Additional waiting in OR" events were purposefully excluded from our timeline. Simulated patients transition through three major stages: (1) preoperative patient preparation; (2) OR occupation (surgical time); and (3) OR preparation (turnover). The patient leaves the OR immediately upon skin closure, instantly triggering the OR preparation phase for the subsequent case.

**Model Inputs**

1. *Surgery type and operative time*

Operative times for each surgery type were simulated using truncated lognormal distributions. The distributions were parameterized utilizing the empirical mean and standard deviation (SD) derived from the real-world dataset, and truncated by the historical minimum and maximum values to prevent mathematically implausible extremes. For instances where only a single case existed for a specific procedure (SD = 0), the operative time was reverted to a constant mean value to prevent simulation deadlock.

1. *Other workflow parameters*

Parameters governing the processing times of the remaining workflow stages were derived from structured clinical interviews and historical guidelines. OR preparation (turnover) time was modeled as a triangular distribution. In the historical baseline and regular slow-track OR scenarios, this was parameterized as Triangle (min=25, mode=27.5, max=30) minutes. In the optimized centralized fast-track pool scenario, the homogeneous nature of the procedures compresses the OR preparation time to Triangle (min=10, mode=12.5, max=15) minutes.

1. *Estimation of the daily work time limit*

Due to strict workforce management policies, ORs cannot operate indefinitely. The model implements a hard cut-off limit (t_limit≤600minutes) for initiating new surgeries.

1. *Baseline Calibration*

In reality, the exact cut-off time fluctuates based on human heuristic decisions (e.g., surgeons refusing to start complex cases near shift-end). To prevent the systematic capacity overestimation inherent to static averaging formulas, we utilized a Retrospective Closed-loop Iterative Calibration. The daily cut-off limit for the baseline model was iteratively fine-tuned until the simulated throughput matched the historical real-world throughput with a negligible error margin (< 2%).

1. *Prospective Dynamic Safety-Buffer Policy*

For the scenario analysis involving segregated fast-track and slow-track ORs, historical calibration was impossible as these represented novel, unobserved systems. We introduced a Dynamic Safety-Buffer Policy, calculating independent cut-off limits based on expected workloads: t_limit=600-[E(Time surgery)+E(Time turnover)]. The primary optimized scenario used fast-track OR preparation Triangle(10, 12.5, 15) minutes versus regular-OR Triangle(25, 27.5, 30) minutes, corresponding to a 15-minute mean turnover reduction. Conservative 5- and 10-minute reduction scenarios were analyzed separately.

This scientifically replicates clinical behavior: highly homogeneous fast-track surgeries permit admissions much closer to the 600-minute mark, whereas complex slow-track surgeries enforce a much earlier cut-off to buffer against severe overtime risks.

**Model verification and validation**

The discrete-event simulation framework was engineered in R 4.6.0 using the simmer package 4.4.7. Verification and validation were executed in two stages. Initially, the structural logic and parameter assumptions were corroborated via clinical consultation with the head OR nurse. Subsequently, quantitative validity was achieved by running the baseline model for 1,000 iterations per department. The model was deemed robust when the variance between the simulated aggregate throughput and the actual real-world surgical volume converged to less than 2% across all nine departments.

**Scenario Analysis**

Rather than evaluating simple parameter reductions (e.g., adding new surgical equipment), our scenario analysis explored complex, macro-level operational restructuring.

To resolve the "Capacity Fragmentation Penalty" observed when segregating fast-track procedures within small, isolated departments, we simulated an Asymmetric Centralized Pooling strategy. The optimization algorithm operated via a two-step framework:

(1) Prospective Simulation Probing (PSP) & SLA Guarantee: A 2,000-iteration DES probe was deployed to determine the true, variance-penalized daily capacity for complex slow-track surgeries. Each department was then allocated the number of regular OR days required to reproduce its historical severe-case volume (Service Level Agreement floor). PSP capacity distributions are reported in Table S2.

(2) Greedy Bottleneck Optimization: All remaining OR days across the hospital were surrendered to a centralized fast-track pool. To preserve clinical diversity, a limiting-factor proportional scaling constraint was evaluated as lambda = min(lambda_fast, lambda_slow), where lambda_fast is the simulated fast-track output relative to historical fast-track volume and lambda_slow is the protected slow-track output relative to historical slow-track volume. Allocations with lambda_slow <1 were rejected, and local departmental segregation was screened against each department's baseline throughput.

**Table S1** Model Input

| Surgery | Patient N | Patient % | Distribution | Mean | SD | Min | Max | Data Source |
| --- | --- | --- | --- | --- | --- | --- | --- | --- |
| Burn Surgery | 122 |  |  |  |  |  |  |  |
| Complicated flap reconstruction | 6 | 4.92% | Truncated lognormal | 125.83 | 33.97 | 90 | 175 | Real data |
| Debridement | 13 | 10.66% | Truncated lognormal | 30.77 | 21.97 | 5 | 80 | Real data |
| Scar contracture release | 32 | 26.23% | Truncated lognormal | 9.69 | 7.06 | 6 | 40 | Real data |
| Skin flap transplantation | 63 | 51.64% | Truncated lognormal | 30.24 | 29.41 | 7 | 165 | Real data |
| other | 8 | 6.56% | Truncated lognormal | 51.25 | 23.26 | 25 | 95 | Real data |
| Dermatology | 107 |  |  |  |  |  |  |  |
| Skin flap transplantation | 7 | 6.54% | Truncated lognormal | 38.57 | 15.2 | 25 | 65 | Real data |
| Skin lesion excision | 28 | 26.17% | Truncated lognormal | 18.21 | 8.19 | 5 | 35 | Real data |
| Skin lesion excision and Flap graft repair | 72 | 67.29% | Truncated lognormal | 49.93 | 22.93 | 20 | 160 | Real data |
| ENT | 442 |  |  |  |  |  |  |  |
| Adenoidectomy | 13 | 2.94% | Truncated lognormal | 12.85 | 7.66 | 5 | 25 | Real data |
| Cochlear implantation | 3 | 0.68% | Truncated lognormal | 170 | 47.7 | 140 | 225 | Real data |
| Pharyngolaryngeal Lesion excision | 8 | 1.81% | Truncated lognormal | 41.25 | 12.75 | 20 | 55 | Real data |
| Uvulopalatopharyngoplasty | 375 | 84.84% | Truncated lognormal | 13.99 | 9.25 | 5 | 75 | Real data |
| Uvulopalatopharyngoplasty+Adenoidectomy | 20 | 4.52% | Truncated lognormal | 51.75 | 14.35 | 15 | 75 | Real data |
| other | 23 | 5.20% | Truncated lognormal | 39.87 | 19.47 | 5 | 95 | Real data |
| General Surgery | 288 |  |  |  |  |  |  |  |
| Appendectomy | 41 | 14.24% | Truncated lognormal | 82.93 | 24.6 | 45 | 130 | Real data |
| Branchial cleft fistula excision | 3 | 1.04% | Truncated lognormal | 26.67 | 10.41 | 15 | 35 | Real data |
| Hernia repair | 69 | 23.96% | Truncated lognormal | 34.35 | 15.12 | 10 | 95 | Real data |
| Intestinal surgery | 18 | 6.25% | Truncated lognormal | 161.67 | 93.15 | 65 | 420 | Real data |
| Labial Adhesion Surgery | 26 | 9.03% | Truncated lognormal | 5.38 | 1.36 | 5 | 10 | Real data |
| Lymphadenectomy | 9 | 3.12% | Truncated lognormal | 31.11 | 11.93 | 10 | 50 | Real data |
| Oophorectomy | 13 | 4.51% | Truncated lognormal | 107.69 | 37.28 | 60 | 205 | Real data |
| Sclerotherapy for lymphatic malformation | 10 | 3.47% | Truncated lognormal | 18 | 8.56 | 5 | 30 | Real data |
| Soft tissue lesion excision | 63 | 21.88% | Truncated lognormal | 35.16 | 17.34 | 5 | 80 | Real data |
| Urachal cyst excision | 20 | 6.94% | Truncated lognormal | 8.25 | 4.38 | 6 | 20 | Real data |
| other | 16 | 5.56% | Truncated lognormal | 118.44 | 107.81 | 7 | 295 | Real data |
| Neurosurgery | 133 |  |  |  |  |  |  |  |
| Biopsy | 8 | 6.02% | Truncated lognormal | 24.38 | 6.78 | 20 | 40 | Real data |
| Brain lesion excision | 25 | 18.80% | Truncated lognormal | 134.2 | 131.46 | 10 | 610 | Real data |
| Craniosynostosis surgery | 18 | 13.53% | Truncated lognormal | 127.5 | 43.63 | 50 | 210 | Real data |
| Craniotomy and hematoma evacuation | 4 | 3.01% | Truncated lognormal | 101.25 | 48.54 | 40 | 145 | Real data |
| Hematoma drainage | 10 | 7.52% | Truncated lognormal | 14 | 9.94 | 5 | 35 | Real data |
| Spinal cord lesion resection | 35 | 26.32% | Truncated lognormal | 111.86 | 90.85 | 25 | 325 | Real data |
| other | 15 | 11.28% | Truncated lognormal | 113.67 | 94.01 | 30 | 310 | Real data |
| ventriculoperitoneal shunt | 18 | 13.53% | Truncated lognormal | 83.89 | 36.84 | 35 | 180 | Real data |
| OMFS | 96 |  |  |  |  |  |  |  |

Table S1（continued）

| Alveolar cleft bone grafting | 4 | 4.17% | Truncated lognormal | 68.75 | 20.16 | 40 | 85 | Real data |
| --- | --- | --- | --- | --- | --- | --- | --- | --- |
| Cheiloplasty | 5 | 5.21% | Truncated lognormal | 71 | 21.91 | 40 | 100 | Real data |
| Cleft palate repair | 11 | 11.46% | Truncated lognormal | 45 | 15.97 | 20 | 70 | Real data |
| Dental filling | 18 | 18.75% | Truncated lognormal | 83.33 | 19.4 | 50 | 115 | Real data |
| Impacted tooth extraction | 33 | 34.38% | Truncated lognormal | 46.06 | 22.73 | 10 | 95 | Real data |
| Lingual frenulum lengthening | 15 | 15.62% | Truncated lognormal | 11.67 | 6.45 | 5 | 30 | Real data |
| Resection of jaw cyst | 5 | 5.21% | Truncated lognormal | 38 | 13.04 | 25 | 55 | Real data |
| other | 5 | 5.21% | Truncated lognormal | 44 | 18.51 | 30 | 75 | Real data |
| Ophthalmology | 218 |  |  |  |  |  |  |  |
| Chalazion curettage | 45 | 20.64% | Truncated lognormal | 12.89 | 7.57 | 5 | 35 | Real data |
| Entropion Correction with Eyelid Reconstruction | 55 | 25.23% | Truncated lognormal | 60.27 | 17.68 | 20 | 95 | Real data |
| Levator muscle shortening for ptosis | 6 | 2.75% | Truncated lognormal | 41.67 | 16.33 | 30 | 65 | Real data |
| Nasolacrimal Duct Probing | 4 | 1.83% | Truncated lognormal | 21.25 | 22.87 | 5 | 55 | Real data |
| Strabismus surgery | 97 | 44.50% | Truncated lognormal | 33.35 | 13.97 | 5 | 85 | Real data |
| other | 11 | 5.05% | Truncated lognormal | 40 | 28.37 | 5 | 90 | Real data |
| Orthopedics | 433 |  |  |  |  |  |  |  |
| Arthroscopic repair | 24 | 5.54% | Truncated lognormal | 103.96 | 53.18 | 30 | 274 | Real data |
| Bone lesion excision | 29 | 6.70% | Truncated lognormal | 105.52 | 61.23 | 20 | 280 | Real data |
| Complex internal fixation removal | 65 | 15.01% | Truncated lognormal | 56.54 | 38.74 | 15 | 195 | Real data |
| Debridement | 79 | 18.24% | Truncated lognormal | 29.78 | 26.14 | 5 | 170 | Real data |
| Internal fixation surgery | 90 | 20.79% | Truncated lognormal | 53.83 | 62.32 | 10 | 450 | Real data |
| Quick internal fixation removal | 116 | 26.79% | Truncated lognormal | 9.09 | 5.46 | 4 | 30 | Real data |
| other | 30 | 6.93% | Truncated lognormal | 100.67 | 156.92 | 5 | 870 | Real data |
| Urology | 756 |  |  |  |  |  |  |  |
| Circumcision | 433 | 57.28% | Truncated lognormal | 6.03 | 3.34 | 5 | 28 | Real data |
| Cystoscopy/Retrograde Urography | 19 | 2.51% | Truncated lognormal | 59.74 | 25.74 | 20 | 105 | Real data |
| Hypospadias repair | 9 | 1.19% | Truncated lognormal | 94.44 | 31.47 | 55 | 140 | Real data |
| Orchiopexy | 20 | 2.65% | Truncated lognormal | 47.25 | 24.52 | 15 | 105 | Real data |
| Penile elongation surgery | 151 | 19.97% | Truncated lognormal | 30.33 | 13.7 | 5 | 90 | Real data |
| Processus Vaginalis ligation | 57 | 7.54% | Truncated lognormal | 24.82 | 14.45 | 5 | 75 | Real data |
| Scrotal exploration | 7 | 0.93% | Truncated lognormal | 19.29 | 10.58 | 5 | 35 | Real data |
| Spermatic vein ligation | 7 | 0.93% | Truncated lognormal | 50 | 20 | 20 | 85 | Real data |
| Ureteral stent removal | 10 | 1.32% | Truncated lognormal | 15 | 12.02 | 5 | 45 | Real data |
| Uroplasty | 11 | 1.46% | Truncated lognormal | 165.91 | 39.93 | 114 | 250 | Real data |
| other | 32 | 4.23% | Truncated lognormal | 38.91 | 45.04 | 5 | 205 | Real data |
| **Other parameters** |  |  |  | **Mode** | / | **min** | **max** |  |
| Preoperative patient preparation | / | 100% | Triangle | 20 | / | 15 | 60 | Head nurse |
|  | / | 100% | Triangle | 27.5 | / | 25 | 30 | Head nurse |
|  | / |  |  | **Constant** | / | / | / |  |
|  |  |  | Constant | 20 | / | / | / |  |

Source-data audit note: the original supplementary table incorrectly listed neurosurgery biopsy maximum duration as 140 minutes. The audited raw OR log contains eight biopsy cases with a maximum of 40 minutes; this table has been corrected accordingly.

**Table S2. PSP slow-track daily capacity estimates used for SLA-floor allocation.**

| **Department** | **Slow baseline** | **Daily mean (SD)** | **Median** | **95% SI** | **Retained slow OR-days** |
| --- | --- | --- | --- | --- | --- |
| Burn Surgery | 90 | 8.76 (1.46) | 9 | 6-11 | 11 |
| Dermatology | 79 | 7.29 (0.77) | 7 | 6-9 | 11 |
| ENT | 54 | 7.32 (1.11) | 8 | 5-9 | 8 |
| General | 229 | 6.44 (1.34) | 6 | 4-9 | 36 |
| Neurosurgery | 115 | 4.01 (0.92) | 4 | 2-6 | 29 |
| OMFS | 81 | 6.87 (0.76) | 7 | 6-8 | 12 |
| Ophthalmology | 173 | 8.25 (0.80) | 8 | 7-10 | 21 |
| Orthopedics | 317 | 6.47 (1.51) | 7 | 3-9 | 49 |
| Urology | 323 | 8.79 (1.36) | 9 | 6-11 | 37 |

**Table S3. Edge-case fast-track classification sensitivity analysis.**

| **Scenario** | **Slow baseline fixed** | **Fast-track mean (SD)** | **Fast-track median** | **Fast-track 95% SI** | **Total mean (SD)** | **Increase** |
| --- | --- | --- | --- | --- | --- | --- |
| Primary fast-track set | 1461 | 2127.7 (13.5) | 2128 | 2101-2153 | 3588.7 (13.5) | 38.29% |
| Edge-case procedures excluded | 1510 | 2158.9 (13.3) | 2159 | 2132-2184 | 3668.9 (13.3) | 41.38% |
